# Impact of high-dose pelvic radiotherapy combined with chemotherapy on local control, symptom relief, and safety in patients with stage IVB cervical cancer (FIGO 2018): a two-center retrospective study

**DOI:** 10.1007/s11604-025-01923-1

**Published:** 2025-12-24

**Authors:** Takaaki Nakashima, Keiji Matsumoto, Tadamasa Yoshitake, Naonobu Kunitake, Madoka Abe, Kazuya Ariyoshi, Hideaki Yahata, Kousei Ishigami

**Affiliations:** 1 Department of Radiation Oncology, National Kyushu Cancer Center, 3-1-1 Notame, Minami-Ku, Fukuoka, 811-1395 Japan; 2 Department of Clinical Radiology, Graduate School of Medical Sciences, Kyushu University, Fukuoka, Japan; 3 Department of Gynecology, National Kyushu Cancer Center, Fukuoka, Japan; 4 Department of Obstetrics and Gynecology, Graduate School of Medical Sciences, Kyushu University, Fukuoka, Japan

**Keywords:** Cervical cancer, Stage IVB of 2018 FIGO staging system, Pelvic radiotherapy, Bevacizumab

## Abstract

**Purpose:**

This study evaluated the efficacy and safety of high-dose pelvic radiotherapy combined with chemotherapy, including bevacizumab and immune checkpoint inhibitors (ICI) in patients with stage IVB cervical cancer (CC) based on the 2018 International Federation of Gynecology and Obstetrics (FIGO) cervical cancer staging system.

**Materials and methods:**

A retrospective analysis was conducted on 38 patients with stage IVB CC, as classified by the 2018 FIGO cervical cancer staging system, who received pelvic external beam radiotherapy (≥ 40 Gy) with or without brachytherapy and chemotherapy. Data were collected from two centers. The 2-year local control (LC), progression-free survival (PFS), and overall survival (OS) rates were analyzed using the Kaplan–Meier method. Symptom relief, including reductions in genital bleeding and pain from the primary lesion, was assessed. Acute and late adverse events were also evaluated.

**Results:**

The median follow-up period was 17.5 months. The 2-year LC, PFS, and OS were 82%, 11%, and 47%, respectively. Although the evaluation method has limitations, most patients with genital bleeding and pain from the primary lesion showed improvement in symptoms. Late adverse events of grade ≥ 2 related to both pelvic radiotherapy and bevacizumab included one case of grade 3 gastrointestinal bleeding and two cases of grade 2 fistula.

**Conclusion:**

This two-center study demonstrated that high-dose pelvic radiotherapy combined with chemotherapy, including bevacizumab and ICI, may achieve favorable local control and symptom relief in patients with stage IVB CC while maintaining an acceptable safety profile.

## Introduction

Cervical cancer (CC) that has metastasized to distant organs is classified as stage IVB according to the 2018 International Federation of Gynecology and Obstetrics (FIGO) cervical cancer staging system, accounting for approximately 10% of cases [1]. Recently, the phase III, double-blind KEYNOTE-826 trial, which included stage IVB CC cases, demonstrated that first-line pembrolizumab combined with platinum-based chemotherapy, with or without bevacizumab, improved survival outcomes and achieved a high objective response rate [2]. However, median progression-free survival (PFS) in the all-comer population of the pembrolizumab-chemotherapy group was 10.4 months, with a 12-month PFS rate of 44.7%, indicating a persistently poor prognosis, and the analysis for PFS also demonstrated that hazard ratio in the subgroup of patients with metastatic disease at diagnosis was 0.86 (0.62–1.21). Hence, it is essentially insufficient to treat stage IVB CC cases with only chemotherapy even containing immune checkpoint inhibitors (ICIs), and another therapeutic strategy is inevitably required.

According to the National Comprehensive Cancer Network (NCCN) guidelines (version 3. 2024), in addition to systemic therapy and/or best supportive care, local treatment, including individualized external beam radiotherapy (EBRT) with or without concurrent platinum-containing chemotherapy, may also be considered for patients with stage IVB CC who are amenable to local treatment [3]. Regarding local therapy in patients diagnosed with stage IVB CC, the European Society of Gynecological Oncology (ESGO), in collaboration with the European Society for Radiotherapy and Oncology (ESTRO) and the European Society of Pathology (ESP) guidelines for managing patients with CC propose that additional radical pelvic radiotherapy (including image-guided brachytherapy [IGBT] in selected cases) may be considered for patients who have responded to systemic therapy [4]. A large retrospective cohort study demonstrated that definitive pelvic radiotherapy might provide a survival benefit for patients with metastatic CC [5]. However, this study included patients with clinically heterogeneous characteristics and was limited to those with para-aortic node metastasis classified as stage IIIC2 under the 2018 FIGO staging system. In Japan, only a few retrospective studies have suggested a potential survival benefit of combining definitive pelvic radiotherapy with chemotherapy in patients with stage IVB CC according to the 2018 FIGO staging system [6, 7]. To date, no prospective studies have established the optimal treatment indications or efficacy of definitive pelvic radiotherapy for patients with stage IVB CC.

Furthermore, patients with stage IVB CC often receive chemotherapy plus bevacizumab during the clinical course, which increases the risk of vascular complications, including gastrointestinal ulceration and fistula formation, when combined with pelvic radiotherapy [8]. Concerning the interaction between ICIs and radiotherapy, the recent randomized phase 3 trial demonstrated that pembrolizumab with chemoradiotherapy followed by pembrolizumab provided similar rates of common treatment-related adverse events compared with placebo with chemoradiotherapy followed by placebo for locally advanced CC except patients with stage IVB (FIGO 2018) [9]. Few studies have demonstrated the safety of combining pelvic radiotherapy with systemic therapies, such as bevacizumab and ICIs, in patients with stage IVB CC.

In addition to poor prognosis, patients with advanced CC may experience various distressing physical symptoms, including pain, bleeding, hydronephrosis, renal dysfunction, and fistulas, all of which significantly reduce the quality of life. The previous studies of various palliative pelvic radiotherapy revealed the efficacy in controlling the symptoms, such as genital bleeding, pain [10]. No studies demonstrated the degree of efficacy for these serious symptoms in detail that novel chemotherapy and high-dose pelvic radiotherapy combined with systemic therapy could achieve in patients with stage IVB CC of the 2018 FIGO staging system. Not only prolonged survival but sufficient relief of serious intrapelvic symptoms is the essential issue for patients with stage IVB CC. Improvement of intrapelvic disease control with high-dose pelvic radiotherapy might provide durable palliation of intrapelvic symptoms.

This retrospective study aimed to evaluate the survival outcome and symptom relief associated with intrapelvic lesions, and safety of high-dose pelvic radiotherapy combined with systemic therapy, including bevacizumab and ICIs, in Japanese patients with stage IVB CC, according to the 2018 FIGO staging system.

## Materials and methods

### Patients

This retrospective study was conducted at two centers (the National Hospital Organization Kyushu Cancer Center and Kyushu University Hospital) and was approved by each institution’s ethical review board. A total of 951 patients with CC including 103 patients with stage IVB, as classified by the 2018 FIGO staging system, received radiotherapy and the study finally included 38 patients with stage IVB CC who received pelvic EBRT (≥ 40 Gy) and chemotherapy between April 2014 and December 2023. Patients who received only palliative pelvic EBRT (< 40 Gy) or could not be monitored for at least 6 months from EBRT initiation were excluded. Patients treated before the publication of the 2018 FIGO staging system were reclassified according to the 2018 FIGO staging system.

### Radiotherapy

The pelvic EBRT regimen and field were determined individually at both centers. EBRT was administered to either the small pelvis or whole pelvis (with or without an extended field) using 3-dimensional conformal radiotherapy. Treatment was delivered via a linear accelerator (TrueBeam or TrueBeam STx, Varian Medical Systems, Palo Alto, CA; or ONCOR Impression Plus Medical Accelerator, Siemens Healthineers, Erlangen, Germany) with 10-MV photons. For small pelvis EBRT, the clinical target volume (CTV) included the gross tumor volume (GTV) of the primary lesion and adjacent lymph node metastases. The irradiated volume was smaller than that of whole pelvis EBRT, including bilateral common iliac nodes. Extended-field radiotherapy also included para-aortic lymph node metastases. In patients undergoing brachytherapy presented at Table 1, tandem and ovoid applicators were used with a ^192^Iridium remote afterloading system (RALS, Flexitron HDR™ or MicroSelectron HDR™, Elekta AB, Stockholm, Sweden). In cases of extensive genital infiltration or a narrow genital canal, a tandem cylinder applicator was used instead. Brachytherapy planning was based on either 2-dimensional planning or 3-dimensional IGBT (3D-IGBT) using computed tomography (CT) images in Oncentra^®^ (Elekta AB, Stockholm, Sweden). Applicators were fixed with or without interstitial needles, guided by magnetic resonance imaging (MRI) obtained before radiotherapy initiation. Brachytherapy was administered once or twice weekly, with a total of two to four sessions conducted based on the total dose limits for the organs at risk (OARs). For cases utilizing CT-based IGBT, high-risk CTV (HR-CTV) and OARs were delineated according to the guidelines of the Japanese Radiation Oncology Study Group (JROSG) [11]. The relevant OARs included the rectum, sigmoid colon, small intestine, and bladder. The dosing regimen aimed to deliver a dose of more than 6 Gy to the HR-CTV D90 (the dose covering at least 90% of the HR-CTV) at each session while simultaneously restricting the total dose delivered to the OAR D2 cm^3^ (the dose at 2 cm^3^ of the OARs) to less than 70 Gy for the rectum, sigmoid colon, and small intestine and less than 80 Gy for the bladder. The total doses from EBRT and brachytherapy were calculated using the biologically equivalent dose in 2-Gy fractions (EQD2) based on the linear-quadratic (LQ) model. For the central shielding technique, the EBRT dose was excluded. The value of α/β ratio was assumed to be 10 Gy for tumors and 3 Gy for OARs, following the guidelines of the Gynecological Groupe Européen de Curiethérapie-European Society for Radiotherapy and Oncology Study Group (GYN GEC-ESTRO) [12].

**Table 1:** Patient characteristics

	*n* = 38
Age (median, range) (years)	54 (36–83)
ECOG-PS	
0	4
1	25
2	6
3	3
Histology	
Squamous cell carcinoma	24
Adenocarcinoma / adenosquamous carcinoma	11
Other	3
Metastatic sites	
Distant lymph node alone	10
Visceral	28
Tumor size of primary lesion (median, range) (cm)	6 (3–10)
T stage	
1	5
2b	6
3b	16
4	11
N stage	
0	3
1	11
2	24
Hydronephrosis	
Yes	16
No	22
Genital bleeding	
Yes	35
No	3
Pain from primary lesion	
Yes	24
No	14

*ECOG-PS*, eastern cooperative oncology group-performance status

### Statistical analysis

Local control (LC) of the primary lesion, PFS, and overall survival (OS) were calculated from the initiation of pelvic radiotherapy using the Kaplan–Meier method. Prognostic factors were evaluated through univariate analysis using the log-rank test. However, multivariate analysis was not performed due to the study’s small sample size. A *P*-value of < 0.05 was considered statistically significant. All statistical analyses were performed using EZR, a graphical user interface for R (R Foundation for Statistical Computing) [13]. The treatment response for symptoms and signs related to genital bleeding and local pain from the primary lesion were evaluated. Genital bleeding relief was defined as the improvement or cessation of bleeding, as documented in patient medical records. Pain relief was assessed subjectively, defined as pain reduction without an increase in analgesic use, based on patient medical records, rather than following the International Consensus on Palliative Radiotherapy Endpoints [14]. Recurrence patterns were also assessed. Additionally, acute and late adverse events were evaluated using the National Cancer Institute Common Terminology Criteria for Adverse Events (CTCAE), version 4.0.

## Results

### Patient characteristics

A total of 38 patients were enrolled. The most common histological subtype was squamous cell carcinoma (SCC), found in 24 patients (63%), followed by adenocarcinoma or adenosquamous carcinoma in 11 patients (29%). Three patients (8%) had small cell carcinoma, neuroendocrine carcinoma, or undifferentiated carcinoma. T3 or T4 tumors were present in 27 patients (71%), with a median tumor size of 6 cm (range: 3–10 cm). Sixteen patients (42%) developed hydronephrosis, four of whom required ureteral stent placement or nephrostomy before starting pelvic radiotherapy. Before initiating pelvic radiotherapy, 35 patients (92%) experienced genital bleeding, while 24 (63%) reported local pain from the primary lesion.

### Radiotherapy

Table 2 presents the treatment characteristics. All patients received pelvic EBRT with a median total dose of 50 Gy (range: 40–50.4 Gy) and a fraction size of 1.8–2.5 Gy. The most commonly treated pelvic field was the small pelvis (47%). Only two patients received an additional boost to pelvic lymph node metastases, with total doses up to 51 and 53 Gy, respectively. Thirteen patients (34%) received EBRT for distant metastatic lesions either concurrently with pelvic radiotherapy or after disease progression. The irradiated metastatic sites included the bone (*n* = 7), supraclavicular lymph node (*n* = 4), para-aortic lymph node (*n* = 3), other distant metastatic lymph nodes in the neck, mediastinum, and inguinal regions (*n* = 2), brain (*n* = 2), other visceral organs of liver, lung, and peritoneal dissemination (*n* = 3). Five patients received EBRT concurrently with pelvic radiotherapy for distant metastatic lesions, with doses of 45 Gy delivered in 25 fractions or 50.4 Gy in 28 fractions. Additionally, stereotactic ablative radiotherapy was performed in three patients, though not concurrently with pelvic radiotherapy.

**Table 2:** Treatment characteristics

	*n* = 38
Pelvic EBRT dose (median, range) (Gy)	50 (40–50.4)
Radiation field	
Small pelvis	18
Whole pelvis	9
Extended field	11
Lymph node boost	
Yes	2
No	36
Radiotherapy for distant metastasis	
Yes	13
No	25
Brachytherapy	
Yes	25
No	13
Brachytherapy dose (median, range) (Gy)	18 (12–24)
Initial treatment	
Chemotherapy alone	5
Pelvic radiotherapy	33
Concurrent chemotherapy regimens	
Cisplatin	8
Carboplatin + Paclitaxel	25
Other	3
No concurrent chemotherapy	2
Bevacizumab administration during follow-up	
Yes	19
No	19
ICI administration during follow-up	
Yes	9
No	29

*EBRT*, external beam radiotherapy; *ICI*, immune checkpoint inhibitor

Intracavitary brachytherapy was administered to 25 patients (66%), with a median total dose of 18 Gy (range: 12–24 Gy) over 2–4 sessions. Two patients received brachytherapy using 2-dimensional planning, with a prescribed dose of 6 Gy to point A, according to the Manchester System. In the remaining patients, CT-based 3D-IGBT was performed. In nine patients treated with 3D-IGBT planning according to the JROSG guidelines [11], the median total dose of HR-CTV D90 in EQD2 was 65.8 Gy (range: 55.6–86.0 Gy). The median total OARs D2 cm^3^ doses in EQD2 were 69.0 Gy (range: 61.7–73.6 Gy) to the rectum, 57.7 Gy (range: 49.1–67.8 Gy) to the sigmoid colon, 58.0 Gy (range: 50.0–69.8 Gy) to the small intestine, and 73.1 Gy (range: 60.1–81.3 Gy) to the bladder.

### Chemotherapy

Initially, five patients received chemotherapy alone, followed by pelvic radiotherapy due to disease progression. In the remaining 33 cases, pelvic radiotherapy was administered as the initial treatment. Concurrent chemotherapy was administered to 36 patients (95%), most commonly with paclitaxel (70 mg/m^2^) and carboplatin (area under the curve 2) weekly or cisplatin (40 mg/m^2^) weekly. Subsequent chemotherapy following pelvic radiotherapy was administered to 37 patients (97%). The major regimen, used in 34 patients (89%), consisted of paclitaxel (175 mg/m^2^) and carboplatin (area under the curve 4–6) triweekly. Additionally, 19 patients (50%) received bevacizumab (15 mg/kg), and nine patients (24%) were treated with ICI, either pembrolizumab (200 mg) or cemiplimab (350 mg) triweekly during follow-up. Only one patient did not receive chemotherapy following pelvic chemoradiotherapy.

### Treatment outcomes

**Fig. 1:**
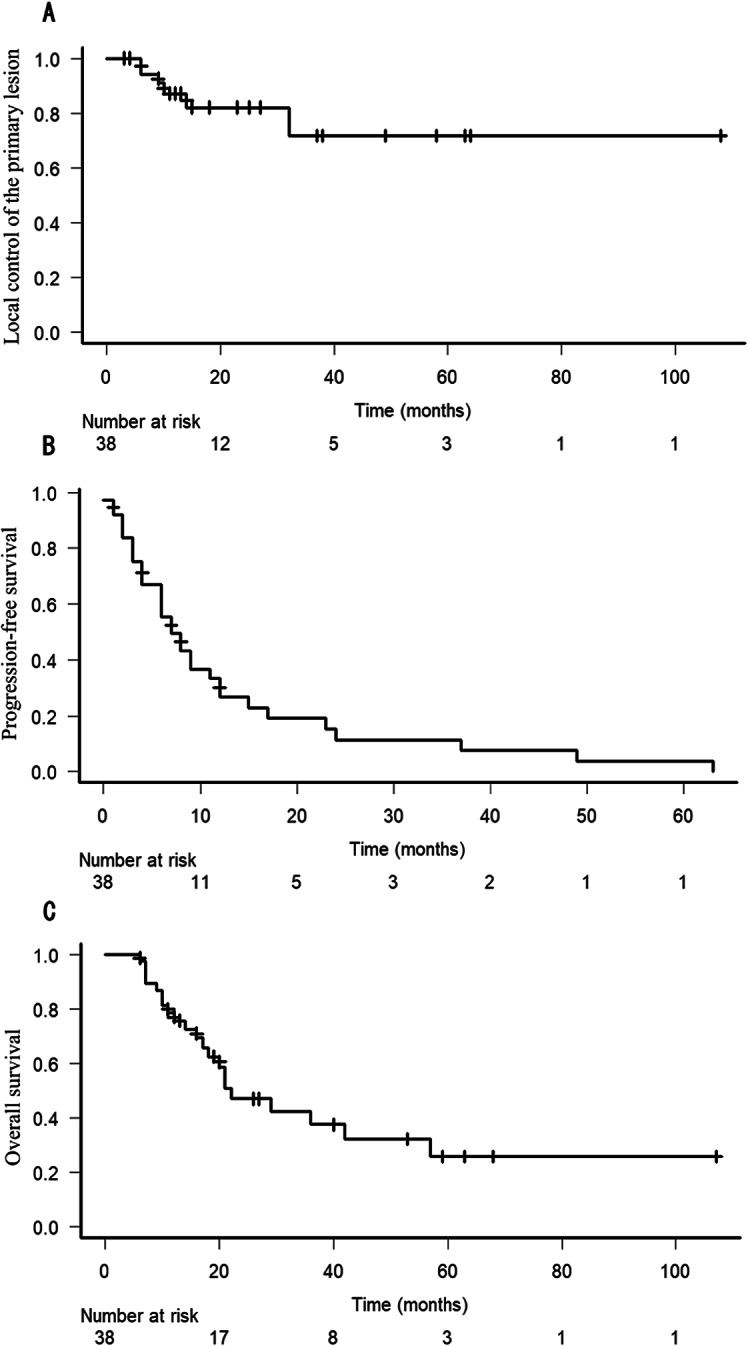
Kaplan–Meier survival curve. **A**: local control (LC) of the primary lesion, **B** progression-free survival (PFS), and **C** overall survival (OS)

**Fig. 2:**
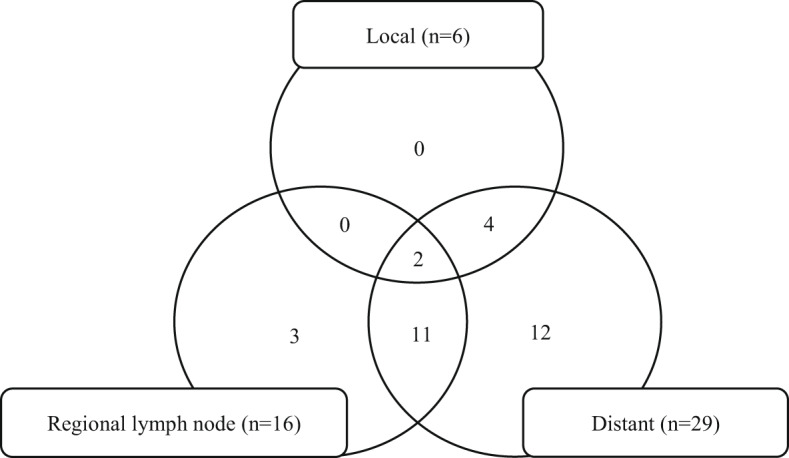
Patterns of progression. The Venn diagram illustrates the distribution of progression patterns

The median follow-up duration was 17.5 months (range: 6–107 months). Thirty-two patients (84%) achieved LC of primary lesion, while 26 of 35 patients (74%) also achieved LC of regional lymph node metastases irradiated concurrently with primary lesion during follow-up. The 2-year LC, PFS, and OS rates were 82%, 11%, and 47%, respectively (Fig. 1). Table 3 presents the patient characteristics of all six patients (16%) who developed local progression of the primary lesion. Notably, all patients with SCC treated with brachytherapy achieved LC of the primary lesion. Among the nine patients with progressive irradiated regional lymph node metastases, three developed clinically significant complications, including cancer-related pain, deep vein thrombosis, pulmonary thromboembolism, hydronephrosis, and fistula formation. Overall, 16 patients (42%) developed regional lymph node progression, while 29 patients (76%) developed distant metastasis progression (Fig. 2).

**Table 3:** Details in cases with local recurrence of primary lesion

Case	Histology	T stage	Tumor size (cm)	Pelvic EBRT dose (Gy)	Brachytherapy (Gy)	Initial treatment	Duration of local control (month)	Events from local progression
1	Non-SCC	2b	6.0	50.4	12	Chemotherapy	14	No
2	Non-SCC	2b	5.5	45	24	Pelvic CRT	6	Bowel obstruction Hydronephrosis
3	SCC	4	4.2	50	0	Pelvic CRT	9	No
4	Non-SCC	3b	7.0	50	0	Pelvic CRT	10	No
5	SCC	4	10.0	45	0	Pelvic CRT	32	No
6	Non-SCC	4	5.0	50	18	Chemotherapy	6	Hydronephrosis

*SCC*, squamous cell carcinoma; *EBRT*, external beam radiotherapy;

*CRT*, chemoradiotherapy

All 35 patients who presented with genital bleeding experienced improvement or cessation of bleeding after pelvic radiotherapy, although anticoagulant or antiplatelet agents were prescribed for 11 patients (31%) during the follow-up. No clinical records confirmed that all six patients with local progression of the primary lesion experienced worsening genital bleeding. Twenty-three (96%) of the 24 patients with local pain from the primary lesion achieved pain reduction without requiring an increase in analgesic use after pelvic radiotherapy, rather than following the International Consensus on Palliative Radiotherapy Endpoints [14].

### Univariate analysis of prognostic factors

In the univariate analysis of prognostic factors, non-squamous histology was the only significantly poor prognostic factor for LC (*P* = 0.047) and PFS (*P* = 0.00679) (Table 4). T stage, primary tumor size before pelvic radiotherapy, and brachytherapy administration were not significantly associated with LC. Additionally, local progression of the primary lesion was not a significant poor prognostic factor for OS.

**Table 4:** Univariate analysis for survival outcomes

Prognostic factor	*n* = 38	LC		PFS		OS	
		2-year (%)	*P*-value	2-year (%)	*P*-value	2-year (%)	*P*-value
Age (year)			0.351		0.0622		0.131
<55	20	81		0		37	
≥55	18	83		21		58	
ECOG-PS			0.738		0.0914		0.558
0–1	29	56		0.7		44	
2–3	9	85		15		56	
Histology			0.047		0.00679		0.0854
SCC	24	95		15		59	
non-SCC	14	59		0		25	
T stage			0.833		0.544		0.462
<3b	11	72		0		56	
≥3b	27	85		14		43	
Tumor size (cm)			0.992		0.195		0.0763
<6	18	80		13		61	
≥6	20	79		0.6		32	
Distant metastasis			0.621		0.438		0.786
Lymph node alone	11	88		13		51	
Visceral	27	80		0.7		44	
Brachytherapy			0.352		0.837		0.652
Yes	25	83		10		45	
No	13	77		0.8		50	
Progression of primary lesion					0.124		0.758
Yes	6			0		62	
No	32			11		45	

*ECOG-PS*, eastern cooperative oncology group-performance status; *SCC*, squamous cell carcinoma

### Toxicities

Table 5 summarizes acute adverse events of grade ≥ 3 and late adverse events of grade ≥ 2. Twenty-four patients (63%) had neutropenia of grade 3 or 4, including three cases of grade 3 febrile neutropenia. Six patients (15%) experienced grade 3 diarrhea, while no patients experienced grade 4 late adverse events. Two patients who developed fistulas received bevacizumab during follow-up. One patient developed a fistula between an irradiated obturator lymph node metastasis and the sigmoid colon. The obturator lymph node metastasis was irradiated with 50.4 Gy in 28 fractions. Following the progression of the disease, the patient underwent chemotherapy including bevacizumab. However, the lymphatic lesion exhibited an increase in size over time, resulting in its invaded of the sigmoid colon. The other patient, whose primary lesion initially invaded the bladder, developed a vesicovaginal fistula without local progression of the primary lesion or grade 3 gastrointestinal bleeding from the rectum. This patient underwent brachytherapy, receiving a total dose in EQD2 of 73.6 Gy to the rectum and 73.5 Gy to the bladder. Clinically significant rectal bleeding occurred approximately 14 months after initiating pelvic radiotherapy (approximately 5 months after initiating bevacizumab treatment), necessitating argon plasma coagulation and transfusion. Four patients developed bowel obstruction due to lymph node metastasis (*n* = 2), primary tumor progression (*n* = 1), and peritoneal dissemination (*n* = 1). Ten patients (26%), including six patients treated with bevacizumab, developed thromboembolism, while four patients (10%), including two patients treated with bevacizumab, developed cerebral infarction. None of the patients experienced enteritis associated with immune-related adverse events of grade ≥ 2.

**Table 5:** Acute (grade ≥ 3) and late (grade ≥ 2) adverse event

Acute adverse event	Grade 3	Grade 4	Grade ≥ 3 (%)
Neutropenia	17	7	24 (63)
Febrile neutropenia	3	0	3 (7)
Anemia	1	0	1 (2)
Thrombocytopenia	4	1	5 (13)
hyponatremia	1	0	1 (2)
Radiation dermatitis	1	0	1 (2)
Acute renal injury	1	0	1 (2)
Anorexia	1	0	1 (2)
Diarrhea	6	0	6 (15)
Urinary infection	1	0	1 (2)
Biliary infection	1	0	1 (2)
Late adverse event	Grade 2	Grade 3	Grade ≥ 2 (%)
Gastrointestinal bleeding	0	1	1 (2)
Bowel obstruction	0	4	4 (10)
Hematuria	0	0	0 (0)
Urinary obstruction	0	3	3 (7)
Fistula	2	0	2 (5)
Thromboembolism	7	3	10 (26)
Cerebral stroke	4	0	4 (10)
Lymphoedema	0	1	1 (2)
Bone fracture	1	0	0 (0)
irAE enteritis	0	0	0 (0)

*irAE*, immune-related adverse event

## Discussion

This study demonstrated a 2-year LC rate of 82%, despite 37% of cases involving non-squamous histology, 71% presenting with advanced T stage (T3 or higher), and brachytherapy being administered in only 66% of cases. Consequently, 32 (84%) and 26 (74%) patients achieved LC of primary lesions and regional lymph node metastases, respectively, within the irradiated field during follow-up. These outcomes are likely attributable to the administration of high-dose EBRT combined with concurrent chemotherapy, followed by subsequent chemotherapy courses. Although few studies have specifically reported on LC of primary lesions and regional lymph nodes in patients with stage IVB CC according to the 2018 FIGO staging system, the outcomes were comparable with those of previous investigations involving high-dose pelvic radiotherapy and chemotherapy for patients with stage IVB CC within the same FIGO classification [7, 15]. However, the LC of primary lesions in this study was lower than that reported in the EMBRACE-I study, which demonstrated 5-year LC rates of 92% and 89% for patients with stage IIIB and IVB CC, respectively, according to the 2009 FIGO staging system [16]. In this study, only 66% of patients received brachytherapy, and the total HR-CTV D90 dose was lower than that in the EMBRACE study. However, a direct comparison of outcomes is challenging due to differences in chemotherapy regimens and the use of IGBT based on MRI in the EMBRACE study.

Most local progression of primary lesions in this study occurred within approximately 1 year of follow-up. One-third of patients with tumor progression of either primary lesions or regional lymph node metastases consequently experienced worsening clinical symptoms, including bowel obstruction, hydronephrosis, and thrombosis, due to the progression of irradiated primary lesions and regional lymph nodes. Although distant metastasis was the most frequent recurrence pattern, as demonstrated in a previous study [15], no patients in our study developed solely local progression of the primary lesion. The EMBRACE-I study demonstrated poor clinical outcomes for patients with local failure, with a median survival of 10 months and a 5-year OS rate of 23% [17]. Another study found that local progression following definitive chemoradiotherapy and IGBT for locally advanced CC led to serious symptoms in 75% of patients [18]. In the Japan Clinical Oncology Group (JCOG) 0505 trial, triweekly paclitaxel and carboplatin demonstrated a median PFS of 6.2 months, with a 1-year PFS rate of 16.5% for patients with metastatic or recurrent CC [19]. Similarly, the KEYNOTE-826 trial showed that approximately half of patients treated with first-line pembrolizumab plus platinum-based chemotherapy, with or without bevacizumab, developed disease progression at 1 year [2]. Although these trials did not explicitly demonstrate LC rates of primary lesions or the efficacy of symptom relief, chemotherapy alone may be insufficient to achieve durable LC of the primary lesion. To improve LC, prevent life-threatening symptoms, and enhance quality of life, pelvic radiotherapy with dose escalation combined with brachytherapy may offer a promising approach.

Our study demonstrated that combining brachytherapy with EBRT did not significantly impact LC, and the progression of the primary lesion was not significantly associated with OS in univariate analysis. Although the significance and definitive indications of high-dose EBRT and brachytherapy for metastatic CC have not been firmly established, several retrospective studies have shown a correlation between dose escalation and brachytherapy with improved oncologic outcomes. For example, Laville et al. conducted a study involving 164 patients, including 39 (23.8%) with distant metastases, who were treated with upfront platinum-based chemotherapy (3–6 cycles), followed by chemoradiation. They found that chemoradiation combined with IGBT achieved a 3-year LC of 90%, with a dose-dependent effect on OS [15]. Yin et al. analyzed 48 patients treated with chemotherapy combined with definitive EBRT and high dose-rate brachytherapy for primary lesions or palliative EBRT for primary pelvic lesions. They showed that chemotherapy combined with definitive EBRT and high dose-rate brachytherapy improved pelvic LC and was an independent prognostic factor associated with reduced mortality and a 60% lower risk of progression [20]. Additionally, they found that the number of brachytherapy sessions and HR-CTV D90 dose were significantly associated with better OS. Wiley et al. reported the outcomes of 29 patients receiving platinum-based chemotherapy and bevacizumab, finding that the definitive radiation group achieved higher OS than the palliative radiation and chemotherapy-only groups [21].

Our study also identified non-squamous histology as a poor prognostic factor for LC and PFS, consistent with previous research [15]. Patients with adenocarcinomas or adenosquamous carcinomas are at a higher risk for local failure and require higher doses to achieve the same therapeutic effects as patients with squamous cell carcinoma [17]. As Laville et al. indicated that 80 Gy or more of HR-CTV D90 dose was significantly better prognostic factor on OS in patients with metastatic CC including 17% of non-squamous cases [15], dose escalation of pelvic radiotherapy might be an option for stage IVB CC cases of non-squamous histology. Furthermore, a study of patients with neuroendocrine metastatic CC demonstrated that the group treated with chemotherapy and definitive radiation had a longer median OS than those treated with chemotherapy alone [22].

Although patients with metastatic CC may experience various distressing physical symptoms associated with the anatomic lesion of their disease, such as bleeding and pain [10], studies have shown that radiotherapy with various dose fractionations can effectively alleviate these symptoms in patients with CC [23]. This study revealed that all patients with bleeding achieved improvement after radiotherapy, despite anticoagulant or antiplatelet agents being prescribed for 11 patients (31%) during follow-up. Despite the poor LC rate observed in patients with non-squamous primary lesions in this study, no evidence indicated that patients with local progression experienced worsening genital bleeding. Additionally, 23 patients (95%) with pain due to primary lesions experienced relief without requiring an increase in analgesic dosage after radiotherapy.

Regarding adverse events, this study demonstrated that grade ≥ 3 acute adverse events, such as neutropenia, thrombocytopenia, and diarrhea, occurred more frequently than those reported in a recent trial of chemoradiotherapy for locally advanced CC [24]. This difference is likely due to the more frequent bone marrow suppression associated with the paclitaxel and carboplatin regimens commonly used in this study compared to the single-agent cisplatin regimens [19, 25]. However, the frequency was comparable to those observed with conventional paclitaxel and carboplatin regimens in the JCOG 0505 trial [19]. A previous Japanese retrospective study involving concurrent radiotherapy with weekly paclitaxel and carboplatin in patients with CC also demonstrated similar rates of neutropenia and diarrhea as acute adverse events [26]. Furthermore, another retrospective study revealed that concurrent platinum doublet chemotherapy with radiotherapy was associated with improved survival outcomes compared to the weekly cisplatin regimen [27]. Regarding the increased risk of diarrhea, aside from the chemotherapy regimen, it is noteworthy that the radiotherapy technique used in this study was 3-dimensional conformal radiotherapy rather than intensity-modulated radiotherapy. Meanwhile, late adverse events such as fistulas and gastrointestinal bleeding potentially associated with both pelvic radiotherapy and bevacizumab were less frequent in this study compared to the Gynecologic Oncology Group 240 trial. This randomized controlled phase 3 trial demonstrated that 15% of patients in the chemotherapy plus bevacizumab group developed fistulas, including 6% with grade 3 fistulas, all of whom had previously undergone radiotherapy [8]. In contrast, in the present study, only 50% of patients received bevacizumab during follow-up, and 66% also underwent brachytherapy. This may explain why clinically significant fistulas and gastrointestinal bleeding were less frequent, despite the inclusion of many cases with locally advanced T stage disease. In the present study, tumor invasion, pelvic radiotherapy, and chemotherapy including bevacizumab may be associated with the development of fistulas in two patients. In one patient who developed grade 3 gastrointestinal bleeding from the rectum, the use of pelvic radiotherapy and bevacizumab may have been associated with rectal bleeding. This is because the patient received relatively high dose in EQD2 of 73.6 Gy to the rectum totally.

This study had several limitations, including its retrospective nature, small sample size, short follow-up period, and heterogeneity in patient characteristics and treatment for both radiotherapy and chemotherapy regimens. In this study, the dose of high-dose pelvic radiotherapy is defined as 40 Gy or more, as the dose frequently administered in palliative settings is usually less than 40 Gy. Examples of such doses include 30 Gy in 10 fractions, 20 Gy in 5 fractions, and 8 Gy in 1 fraction in daily clinical practice. According to the NCCN guidelines (version 3. 2024), in patients with an intact cervix (i.e., those who do not have surgery), the primary tumor and regional lymphatics at risk are typically treated with definitive EBRT to a dose of approximately 45 Gy (40–50 Gy) [3]. The definition is not officially prescribed on any clinical guidelines for the treatment of CC, therefore that may include the risk of bias. Five (13%) patients initially received chemotherapy alone, followed by pelvic radiotherapy. As survival outcomes were calculated from the initiation of pelvic radiotherapy, the risk of potential immortal time bias can be introduced. When interpreting survival outcomes, it is necessary to consider this potential limitation. Regarding analyses of prognostic factors, multivariate analysis was not performed due to the study’s small sample size, so the interpretation of prognostic factors remains limited. Additionally, bevacizumab was administered to only 50% of patients, and only 23% received ICI during the entire follow-up. Furthermore, most patients in this study did not receive induction chemotherapy, making comparisons with previous literature challenging. With regard to the evaluation of symptomatic relief, it was not possible to perform a comprehensive subjective assessment based solely on the preceding clinical records. Consequently, the response to pain and genital bleeding may carry the risk of bias and be overestimated, so caution is needed in interpretation due to the possibility of an overestimation bias. In addition, subsequent research is necessary to quantitatively assess the difference in response to high-dose pelvic radiotherapy and palliative dose radiotherapy. Despite its limitations, this study provides valuable insights into treatment strategies for patients with stage IVB CC according to the 2018 FIGO staging system, as literature demonstrating the outcomes of high-dose pelvic radiotherapy combined with chemotherapy in Japanese patients treated with bevacizumab and ICI during follow-up is sparse.

In conclusion, this study demonstrated that high-dose pelvic radiotherapy combined with chemotherapy, including bevacizumab and ICI, safely achieved promising LC and symptom relief of the primary lesion in Japanese patients with stage IVB CC, as classified by the 2018 FIGO staging system. These findings suggest that this approach may be a viable treatment option for this patient population. Future prospective studies are required to evaluate the optimal indications for high-dose pelvic radiotherapy with chemotherapy in this population.

## Data Availability

The data analyzed during this study are available on reasonable request from the corresponding author. The data are not publicly available due to patient privacy concerns and ethical restrictions.
